# Designing Novel Nanoformulations Targeting Glutamate Transporter Excitatory Amino Acid Transporter 2: Implications in Treating Drug Addiction

**Published:** 2015-07-27

**Authors:** PSS Rao, Murali M. Yallapu, Youssef Sari, Paul B. Fisher, Santosh Kumar

**Affiliations:** 1 Department of Pharmaceutical Sciences, College of Pharmacy, University of Tennessee Health Science Center, Memphis, TN 38163, USA; 2 Department of Pharmacology and Experimental Therapeutics, University of Toledo, College of Pharmacy and Pharmaceutical Sciences, Toledo, OH 43614, USA; 3 Department of Human and Molecular Genetics, VCU Institute of Molecular Medicine, VCU Massey Cancer Center, Virginia Commonwealth University, School of Medicine, Richmond, VA 23298, USA

**Keywords:** Glutamate, EAAT2, Drug addiction, Nanoparticles, Transcytosis

## Abstract

Chronic drug abuse is associated with elevated extracellular glutamate concentration in the brain reward regions. Deficit of glutamate clearance has been identified as a contributing factor that leads to enhanced glutamate concentration following extended drug abuse. Importantly, normalization of glutamate level through induction of glutamate transporter 1 (GLT1)/ excitatory amino acid transporter 2 (EAAT2) expression has been described in several in vivo studies. GLT1 upregulators including ceftriaxone, a beta-lactam antibiotic, have been effective in attenuating drug-seeking and drug-consumption behavior in rodent models. However, potential obstacles toward clinical translation of GLT1 (EAAT2) upregulators as treatment for drug addiction might include poor gastrointestinal absorption, serious peripheral adverse effects, and/or suboptimal CNS concentrations. Given the growing success of nanotechnology in targeting CNS ailments, nanoformulating known GLT1 (EAAT2) upregulators for selective uptake across the blood brain barrier presents an ideal therapeutic approach for treating drug addiction. In this review, we summarize the results obtained with promising GLT1 (EAAT2) inducing compounds in animal models recapitulating drug addiction. Additionally, the various nanoformulations that can be employed for selectively increasing the CNS bioavailability of GLT1 (EAAT2) upregulators are discussed. Finally, the applicability of GLT1 (EAAT2) induction via central delivery of drug-loaded nanoformulations is described.

## Introduction

Substance abuse remains a major global healthcare issue with the incidence of drug addiction on the rise. In the United States, the National Institute on Drug Abuse (NIDA) estimates the annual healthcare costs attributed to drug abuse is in excess of $160 billion. The latest national survey on drug use and health reported that approximately 24 million Americans over the age of 12 years used an illicit drug over the past month [1]. In addition, increased incidence of drug use was observed in populations below the age of 25 years and above the age of 50 years [1]. Overall, tobacco smoking, alcohol use, and other illicit drugs are linked to the deaths of over 550,000 Americans every year.

Addiction, as defined by NIDA, is a chronic, relapsing brain disease that is characterized by compulsive drug seeking and use, despite harmful consequences. Drug addiction is primarily driven by neurochemical changes in areas of the brain associated with sensation of pleasure. Over the past several decades, changes in the mesocorticolimbic dopamine system has received particular attention [2]. For psychostimulant drugs and natural rewards like food, release of dopamine in the nucleus accumbens is known to elicit the reward associated with drug [3]. This effect, in turn, over time is known to facilitate the development of drug dependence. However, the complexity of drug addiction treatment lies in the fact that drugs that are abused are known to interact with several neurotransmitter systems.

A large body of evidence suggests a prominent role of glutamatergic neurotransmission in governing drug addiction behavior [4–6]. Glutamate is the major excitatory neurotransmitter in the central nervous system (CNS) and intake of psychostimulants is known to be associated with enhanced release of glutamate in brain regions, including nucleus accumbens and ventral tegmental area [7,8]. In addition, J Personali NanoMed glutamatergic neuron-driven release of dopamine in specific areas of the brain, striatum for instance, has been implicated in relapse in drug use [9]. Interestingly, in alcoholism, a biphasic effect of alcohol consumption towards extracellular glutamate concentrations has been observed [10]. While acute exposure to alcohol is known to attenuate extracellular glutamate concentration, chronic exposure to alcohol is marked by an increase in extracellular glutamate concentration.

Changes in glutamatergic neurotransmission and extracellular glutamate concentration, as reported with several drugs of abuse, have been associated with an attenuated glutamate clearance from the synaptic cleft [11]. The glutamate transporter 1 (GLT1)/ excitatory amino acid transporter 2 (EAAT2) is responsible for the reuptake of more than 90% glutamate in the CNS [12–14]. Following identification of beta lactams as upregulators of GLT1 and EAAT2 expression [15–17], several studies have evaluated the efficacy of beta lactam antibiotics in attenuating drug-seeking behavior in rodents [18,19]. Facilitating glutamate uptake, by treating with compounds which upregulate GLT1 (EAAT2) expression, in the mesocorticolimbic pathways is hypothesized to curb the glutamate-induced relapse to drug seeking.

While few compounds capable of inducing GLT1 (EAAT2) expression have demonstrated promising results in animal studies, clinical translation for these drugs such as ceftriaxone may be hindered by lack of optimal pharmacokinetic properties when the compound is administered orally. With the emergence of nanoformulations as a selective CNS-delivery tool, application of existing nanoformulation strategies to promising GLT1 upregulators is rationalized to yield effective treatment options for drug addiction. In this review article, we summarize the findings from studies investigating promising GLT1 (EAAT2) upregulators in preclinical drug addiction models. In addition, we discuss the emerging trends in design of CNS-directed nanoformulations. Finally, we evaluate the feasibility of nanoformulating GLT1 (EAAT2) upregulators as putative treatment options for drug addiction.

## Normalizing Glutamatergic Neurotransmission: Known GLT1 Upregulators

Normalizing the synaptic glutamate concentrations, through upregulation of GLT1 expression, has shown promising results in preclinical models of drug addiction [20,21]. The role of glutamatergic neurotransmission and significance of modulating GLT1 (EAAT2) expression in treating alcohol use disorder is well known and has been reviewed previously [22,23]. Following the discovery of beta lactam antibiotics as promising GLT1 (EAAT2) upregulators by Rothstein et al. [15], ceftriaxone has been extensively evaluated for its efficacy in attenuating drug-seeking behavior in rodents. Ceftriaxone-induced GLT1 (EAAT2) upregulation proved to be effective in rodent cocaine [25,26], heroin [11], methamphetamine [27], morphine [28], and nicotine [29].

In addition to ceftriaxone, other beta lactam antibiotics have also shown promising GLT1 (EAAT2) modulatory activity in rodent models for drugs of abuse. Ampicillin, cefazolin, and cefoperazone were recently demonstrated to induce the expression of GLT1 (EAAT2) in nucleus accumbens and prefrontal cortex of alcohol preferring P rats [30]. Importantly, this induction in GLT1 (EAAT2) expression was associated with significantly reduced consumption of alcohol by P rats. Interestingly, Schroeder et al. have identified clavulanic acid, a β lactamase, to possess comparable activity to ceftriaxone in attenuating the effects of morphine [31]. Although presently unclear, the similarity of effects between ceftriaxone and clavulanic acid treatments and the presence of a common pharmacophore (beta lactam ring) suggests GLT1 (EAAT2) upregulation as a putative mechanism of action for clavulanic acid. Whether clavulanic acid exerts its effect on GLT1 (EAAT2) expression in a similar manner as ceftriaxone, i.e., through specific regulatory sites in the EAAT2 promoter [17], or through other mechanisms remains to be determined.

Apart from beta lactams, recent reports have demonstrated the ability of a wide spectrum of compounds to promote upregulation of GLT1 (EAAT2) expression. Ganel et al. reported the ability of neuroimmunophilin compound, GPI-1046, to selectively upregulate the expression of GLT1 (EAAT2) in organotypic cultures and primary astrocytes [32]. In a subsequent study, GPI-1046 was found to be effective in attenuating alcohol consumption in male P rats [33]. This observation was associated with significantly enhanced GLT1 (EAAT2) expression in mesocorticolimbic regions of GPI-1046-treated rats compared to saline-treated control animals. In other studies, interesting results were reported for the glutamate transporter activator (R)-(−)-(5)-methyl-1-nicotinoyl-2-pyrazoline (MS-153) in attenuating morphine dependence [34]. Treatment with MS-153 was also reported to have a significant impact on the reward effects of morphine, methamphetamine, and cocaine in mice [35]. In addition to serving as a glutamate transporter activator, recent studies evaluating the influence of MS-153 on alcohol consumption in P rats have demonstrated this compound to be a significant upregulator of GLT1 (EAAT2) [36,37]. Promising GLT1 (EAAT2)-inducing activity has also been identified for a synthetic series of pyridazine derivatives [38]. A representative lead compound from this series, LDN/ OSU-0212320, was recently reported to display favorable GLT1 (EAAT2) upregulation with minimal toxicity/side effects in murine model [39].

Amongst natural compounds, harmine, a β-carboline alkaloid, was reported to upregulate GLT1 (EAAT2) expression in primary cultures of mouse and human astrocytes [40]. Subsequent studies in rat and invertebrates have confirmed the beneficial effects of harminevia induction of GLT1 expression [41, 42]. Interesting results were also reported for the cannabinoid receptor agonist, WIN 55,212-2. Prenatal exposure to WIN 55,212-2 resulted in enhanced expression of GLT1 in frontal cerebral cortex of adolescent rats [43]. In a mouse model for multiple sclerosis, chronic treatment with WIN 55,212-2 was associated with enhanced GLT1 gene expression in spinal cord of treated mice [44]. Further studies are required to define the mechanism by which WIN 55, 212-2 enhances GLT1 (EAAT2) expression in the spinal cord of treated mice. GLT1 modulatory activities have been observed with other Food and Drug Administration (FDA) approved drugs as well. For instance, the tricyclic antidepressant amitriptyline has demonstrated promising effects on the expression of glutamate transporters. Studies conducted in morphine-tolerant rats confirmed amitriptyline-mediated upregulation of GLT1 and glutamate-aspartate transporter (GLAST) expressions [45,46]. In agreement with results obtained with ceftriaxone [19], amitriptyline-induced enhanced expression of glutamate transporters was also confirmed to be mediated through a nuclear transcription factor-kappaB (NFκB)-dependent pathway [47]. Another FDA approved drug, riluzole, for treatment of amyotrophic lateral sclerosis (ALS), was reported to enhance the activity of several glutamate transporters, including GLT1 [48]. Recent studies have extended the effects of riluzole to include the upregulation of GLT1 expression following *in vivo* and *in vitro* treatments with riluzole [49,50].

Overall, following detailed pharmacological studies for safety and efficacy, these compounds can be rationalized to attenuate drug-seeking behavior and prevent relapse of drug use through selective upregulation of GLT1 (EAAT2) expression in synaptic clefts across the mesocorticolimbic pathway. Re-establishing glutamate homeostasis is expected to diminish the reward associated with drugs of abuse and therefore provide beneficial outcomes in delimiting addictive behavior. Since a predominant means of upregulating glutamate transport is through induction of enhanced GLT1 / EAAT2 promoter activity [15–17], approaches employing medium and high throughput screening strategies using the EAAT2 promoter linked to a reporter gene (such as luciferase) that are stably or transiently expressed in appropriate astrocytic cells offers significant potential for identifying novel, safe, and physiologically effective regulators of glutamate activity [17]. These unique agents could have profound effects on ameliorating addiction to various drugs of abuse [17].

## Enhanced Delivery of Drugs Across the Blood Brain Barrier: Application of Nanoformulations

While *in vivo* studies clearly suggest a potential for remedying the impaired glutamate neurochemistry employing GLT1 (EAAT2) upregulators, the evidence for success of such strategies in treating clinical cases of drug addiction remains limited. Lack of desirable pharmacokinetic properties and/or severe peripheral adverse effects may lead to failure of promising preclinical GLT1 (EAAT2)-inducing compounds in human studies. Ceftriaxone treatment, for instance, was associated with a higher incidence of gastrointestinal adverse effects in a Phase 3 clinical trial compared to placebo [51]. Moreover, owing to poor oral bioavailability of ceftriaxone, this clinical study had to employ administration of drug through a central venous catheter. In addition, as reviewed by Nau et al., factors responsible for limited penetrability of drugs like beta lactam antibiotics across the blood brain barrier (BBB) may include high molecular weight, low lipophilicity, plasma protein binding, and affinity for drug efflux transporters [52].

Recent advances in the design of drug delivery systems have demonstrated the applicability of drug-loaded nanoformulations like liposomes, polymeric nanoparticles, and solid lipid nanoparticles in treating CNS ailments [53]. Among these nanoformulations, polymeric nanoparticles, for instance, can be optimized for selective uptake across BBB by targeting receptor-mediated and adsorptive-mediated transcytosis [54,55]. Employing this rationale, enhanced CNS penetration of transferrin-containing gold nanoparticles, via targeting the transferrin receptor expressed on brain capillary endothelial cells, was reported in mice [56]. These nanoparticles exhibited optimum ability to transcytose across the BBB in the presence of moderate affinity of the transferrin receptors.

Enhanced CNS bioavailability has also been observed following the use of positively charged polymer nanoparticles. Of particular interest is the biodegradable and biocompatible polysaccharide chitosan [57]. Compared to control, CNS delivery of estrogen was found to be significantly higher when formulated as chitosan nanoparticles [58]. Furthermore, significantly higher cerebrospinal fluid (CSF) concentration of estrogen were observed when estrogen-loaded chitosan nanoparticles were dosed intranasally as opposed to intravenous administration in rats. In another study, conjugation of chitosan nanospheres with OX26 monoclonal antibodies targeting the transferrin receptors was observed to enhance the uptake of peptide Z-DEVD-FMK-loaded chitosan nanospheres across the BBB [59].

Liposomes constitute another interesting class of nanoformulations that have been shown to successfully deliver drugs to the brain [60]. Liposomes are made up of one or more phospholipid bilayers and are widely used owing to their versatility to carry hydrophobic, hydrophilic, and amphipathic compounds. As reviewed by Pinzon-Daza et al., CNS targeting of liposomes can be enhanced by modifying their surface properties, thereby facilitating passive transport across the BBB, or by conjugating liposomes with antibody/ligand for active uptake into the brain [61]. Such strategies have yielded promising results in the CNS delivery of liposomes carrying neuroprotective [62] and anti-cancer [63] drugs in animal model.

The emergence of magnetic nanoparticles (MNPs) serves as an additional tool to facilitate the bioavailability of drugs in the CNS [64]. Iron oxide based nanoparticles have shown promising results in achieving magnetically-guided delivery of drugs [65]. Owing to their inherent characteristics such as high magnetic resonance imaging, hyperthermia, and magnetic targeting, the MNPs are stabilized by polymeric layers that can be used to encapsulate drugs. Employing liposome encapsulation, magnetic nanoparticles carrying azidothymidine 5′-triphosphate, for example, exhibited 3-fold higher transmigration across the BBB under the influence of external magnetic field compared to free drug [66]. Additional success towards increasing the transmigration efficiency across BBB was achieved by integrating the magnetic and receptor-mediated approaches. A synergistic increase of 50–100% in transport of nanoparticles across the BBB was demonstrated for transferrin embedded MNPs as compared to magnetic force- or transferrin receptor-mediated transportation alone [67].

Overall, several promising nanoformulation strategies have been developed for improved CNS delivery of drug compounds in the past decade. While obstacles to clinical translation of nanoformulations include the lack of long-term toxicity data for nanomaterials and the absence of standardized nanoformulation parameters, the continuing success of nanoformulations in delivering desirable pharmacokinetic profile in animal models is anticipated to expedite the clinical application of these novel dosage forms.

## Nanoformulating GLT1 (EAAT2) Upregulators: Treating Drug Dependence and Relapse

**Figure 1:**
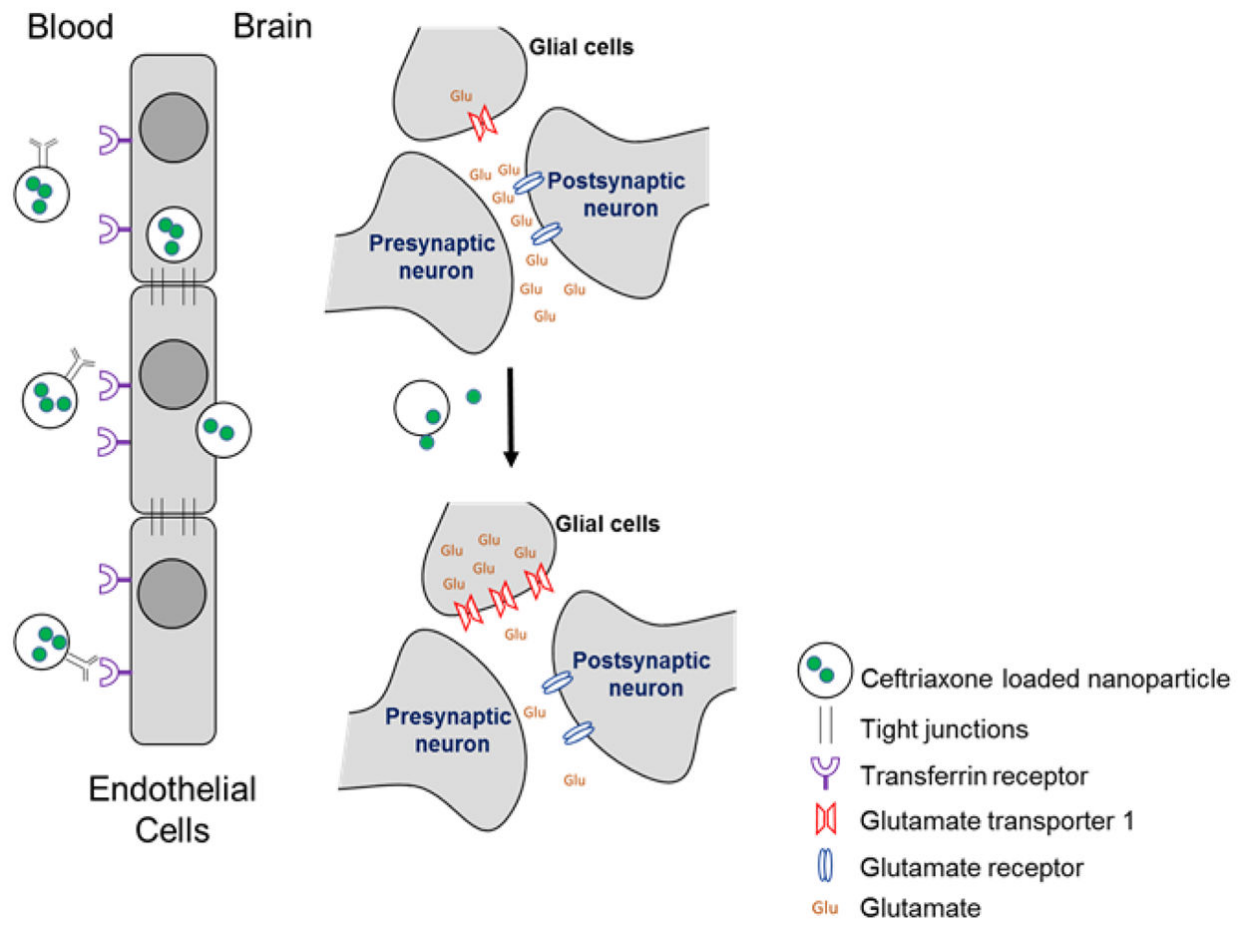
A schematic diagram of proposed mechanism of action for ceftriaxone-loaded nanoparticles. Antibody targeting the transferrin receptors will facilitate the selective uptake of ceftriaxone-loaded nanoparticles across the blood brain barrier. Upon entering the brain, the ceftriaxone released from these nanoparticles will induce the expression of GLT1 (EAAT2) in glial cell. Increased levels of glutamate transporters will facilitate the reuptake of glutamate and decrease the extracellular glutamate concentrations. The resumption of glutamate homeostasis will in principle result in attenuation of drug-seeking behavior.

As described above, re-establishing glutamatergic homeostasis via enhanced expression of GLT1 (EAAT2) has demonstrated satisfactory success in animal models, and represents a potential clinical target for treating drug dependence and relapse. In clinical studies, normalization of glutamatergic neurotransmission can be expected to decrease the reward associated with drug use and facilitate the success of drug rehabilitation programs. While several GLT1 (EAAT2) upregulators discussed in previous section are known to cross the BBB, limited oral bioavailability and/or severe systemic adverse effects may affect the adherence to these medication while treating active drug users. Brain-targeted drug delivery using nanoformulations, on the other hand, can been visioned to eliminate the pharmacokinetic issues when applied to interesting drug candidates. Encapsulating known GLT1 (EAAT2) upregulators like ceftriaxone in liposomes conjugated with surface ligand/antibody targeting the transferrin receptor, for example, can facilitate the transcytosis of drug-loaded liposome across the BBB (Figure 1). Selectively enhancing the central delivery of ceftriaxone can be expected to drastically reduce the daily dosage required for treatment and potentially eliminate the serious hepatobiliary adverse effects that were observed in a recently concluded clinical trial with ceftriaxone [51]. Furthermore, upon reaching the brain, the release of encapsulated ceftriaxone from the nanoformulation would be expected to increase the expression of GLT1 (EAAT2) in astrocytes and facilitate the reuptake of glutamate from the synaptic cleft. The return of extracellular glutamate levels to normal concentrations might also lead to attenuation in drug seeking behavior. Similarly, the use of MNPs to formulate GLT1 (EAAT2) upregulators can potentially enhance the concentration of these drugs in the brain under the influence of an external non-harmful magnetic field.

The innovative strategy of employing nanoformulations to enhance the CNS delivery of GLT1 (EAAT2) upregulators is bolstered by successful applications of such approaches with other classes of drugs, for instance, the antiretroviral therapy for treating neuroAIDS [68]. Substantially higher brain concentration for anti-HIV drugs, that are known to possess poor CNS permeability, has been achieved using nanoformulations. For example, saquinavir (HIV protease inhibitor) and enfuvirtide (HIV fusion inhibitor) have been reported to demonstrate higher brain concentrations following their dosing as nanoformulations [69,70]. Similarly, nanoformulations have yielded encouraging results in treatment of neurodegenerative diseases including amyotrophic lateral sclerosis (ALS), Alzheimer’s disease, and Parkinson’s disease [71,72]. Amongst known GLT1 (EAAT2) upregulators, riluzole loaded solid-lipid nanoparticles designed for selective brain targeting displayed preferential distribution across the CNS as compared to systemic circulation [73]. Selective accumulation in brain, as opposed to peripheral organs, is thought to directly increase the activity in addition to reducing the adverse effects associated with the drug.

Given the flexibility of nanoformulations with respect to their drug carrying capacity, other GLT1 (EAAT2) upregulators such as MS-153 and GPI-1046 can also be packaged in to CNS-targeting nanoformulations to achieve optimum therapeutic results. Moreover, this rationale can be expanded to include multidrug nanoformulations targeting GLT1 (EAAT2) expression in the mesocorticolimbic pathway. Such an approach would enhance the pharmacological response while decreasing both dosage and adverse effects associated with the co-administered drugs. In addition, as reported with antiretroviral drugs [74], the nanoformulated GLT1 (EAAT2) upregulator can also be designed as a long-acting, sustained release formulation. Extending the systemic release of brain-targeting nanoformulation encapsulating a GLT1 (EAAT2) upregulator from a subcutaneous depot could provide a better control over synaptic glutamate concentrations in mesocorticolimbic regions and might prove useful in preventing relapse to drug use. Furthermore, this strategy could potentially eliminate the issue of lack of adherence to daily medication, which is highly prevalent amongst active drug users because of adverse side effects of the drugs.

In conclusion, applications of medium and high throughput screening approaches to identify potentially novel small physiologically active drugs that upregulate GLT1 (EAAT2) expression and nanoformulations of existing GLT1 (EAAT2)-inducing compounds represent novel approaches and an efficient strategy, respectively, for the treatment of drug-dependence and -relapse. Improved delivery of GLT1 (EAAT2) upregulators across the BBB is conceptualized to provide a safe and effective therapeutic option for clinical management of drug use while potentially eliminating the peripheral adverse effects associated with these compounds. Several attempts towards these approaches for better treatment strategy for drug-dependence and -relapse are currently underway.
